# Acknowledgement to Reviewers of *Plants* in 2018

**DOI:** 10.3390/plants8010015

**Published:** 2019-01-09

**Authors:** Plants Editorial Office

**Affiliations:** MDPI, St. Alban-Anlage 66, 4052 Basel, Switzerland

Rigorous peer-review is the corner-stone of high-quality academic publishing. The editorial team greatly appreciates the reviewers who contributed their knowledge and expertise to the journal’s editorial process over the past 12 months. In 2018, a total of 114 papers were published in the journal, with a median time to first decision of 15.4 days and a median time to publication of 37 days. The editors would like to express their sincere gratitude to the following reviewers for their cooperation and dedication in 2018:

Acosta, Ivan F.Leiva-Brondo, MiguelAdamakis, Ioannis-DimosthenisLenhard, MichaelAgnieszka, SzopaLewis, James D.Agudelo-Romero, PatriciaLi, MaoAlba, JuanLi, WanlongAleksandrowicz-Trzcińska, MartaLi, WenyiAlexopoulos, AthanassiosLi, XianranAlhousari, FadiLiao, Pei-ChunAlizadeh, HosseinLibault, MarcAlqudah, AhmedLidon, FernandoAltemimi, AmmarLiolios, Konstantinos A.Álvarez-Fernández, M. AntoniaLionetti, VincenzoAmbrose, ChrisLiu, YongliangAmirkhani, MasoumeLong, YuchenAncuceanu, RobertLongo, RoccoAndreev, KonstantinLu, YouAnwar, RokebulLucarini, MassimoAtes, SerkanLukinavicius, GrazvydasBahmani, RaminLupini, AntonioBai, HuaLütken, HenrikBajgain, RajenMa, GuojiaBaldrich, PatriciaMacnack, NatashaBallizany, WouterMalcolm, BrendonBanilas, GeorgiosManfredini, StefanoBarba, Francisco J.Manjunath, ManuboluBarchenger, DerekMarla, SandeepBarman, ApurbaMartinez, ManuelBarriocanal, CarlesMartínez, SidoniaBarta, CsengeleMartins, NatáliaBazos, IoannisMartos, SoledadBecker, JörgMaruyama-Nakashita, AkikoBenvenuto, EugenioMastrangelo, Anna M.Bhattacharjee, SonaliMatthes, AnnemarieBhowmik, ArnabMattoo, AutarBleckmann, AndreaMavian, CarlaBlein, ThomasMccray, MabryBogdan, IleanaMcCubbin, AndrewBones, AtleMenzel, Christopher M.Bonga, Jan MaxMiguel, MariaBoss, PaulMilella, LuigiBrückner, AdrianMinocha, Subhash C.Brunetti, CeciliaMishra, SasmitaBubici, GiovanniMiyamoto, KojiBunce, JamesMorello, LauraBurghardt, Liana T.Mori, KazuhiroCabot, CatalinaMurai, KojiCaldo, Kristian MarkMurata, JunCampbell, MichaelMurata, ToshihiroCanini, AntonellaMurray, JeremyCao, ZheNachimuthu, GunasekharCapasso, RaffaeleNadia, BazihizinaCardinale, MassimilianoNakka, SrideviCardoso, SusanaNandi, SaikatCarrasco, PedroNeri, DavideCarrubba, AlessandraNotaguchi, MichitakaCarvajal, MicaelaNowicki, MarcinCaser, MatteoOmar, AhmadCazzato, EugenioOrfila, CarolineCeliński, KonradPagnotta, Mario A.Champion, CodyPandolfini, TizianaChang, Hao-XunPanteris, EmmanuelChang, Young KiPappas, Maria L.Chen, DijunPaul, MatthewChen, HaotongPeirats-Llobet, MartaChen, ZhonghuaPentzold, StefanCheng, Juei-TangPereira, Ana LuisaChin, Young-WonPerrine-Walker, FrancineChizzola, RemigiusPetrakis, Panos. V.Çiçek, Serhat SezaiPettolino, FilomenaCiulu, MarcoPezzotti, MarioCommisso, MauroPidatala, Venkata RamanaConstantinidis, TheophanisPollastrini, MartinaContreras, María Del MarProestos, CharalamposCosta, AlexQaderi, MirwaisCrain, JaredQualset, Calvin O.Croce, Anna CletaRai, DilipCullis, ChristopherRamegowda, VenkategowdaDąbrowska, JolantaRanjit, SumanDaras, GerasimosRascio, AgataDavies, JuliaRayon, CatherineDe Bellis, LuigiReed, Kevin F.M.De Diego, NuriaRen, HongDe Mieri, MariaRen, YulinDe Tullio, M.C.Renault, SylvieDel Giudice, RitaRenna, MassimilianoDel Río Celestino, MercedesRicci, AntonellaDeshmukh, RupeshRiechmann, José LuisDevireddy, AmithRodrigo Comino, JesúsDey, PrabuddhaRodríguez-Seijo, AndresDinu-Pîrvu, Cristina ElenaRoh, ChanghyunDong, YiboRoscoe, ThomasDonno, DarioRozhon, WilfriedEichberg, CarstenRubio Valverde, LourdesElbasyoni, IbrahimRuelland, EricElbaum, RivkaSabater, BartolomeEleftheriou, Eleftherios P.Sabella, ErikaEndresen, DagSaito, YoshiroEngelberth, JurgenSalas, José BlancoFagnano, MassimoSantino, AngeloFalistocco, EgiziaSarkar, Saswata SankarFoereid, BenteSato-Nara, KumiFrew, AdamSchäffner, AntonFrugis, GiovannaSchaller, JörgGalindo-González, LeonardoScherf, Katharina A.Gallemí, MarçalSchläppi, Michael R.Gangola, Manu PratapSchwitzguebel, Jean-PaulGao, XiaolongScognamiglio, MonicaGarrido-Cardenas, Jose AntonioSerafini-Fracasini, DanatellaGe, YufengSetzer, William N.Gervasi, TeresaShafian, SanazGhani Zadeh, HosseinShen, JianqiangGianinetti, AlbertoSheng, RuilongGiannakoula, AnastasiaShimoda, HiroshiGiovannini, AnnalisaShrestha, KrishnaGladish, Daniel K.Shunmugam, ArunGniewosz, MałgorzataSilar, PhilippeGombojav, AriunboldSingh, RakshaGrkovic, TanjaSiracusa, LauraGuido, Luis F.Smith, Mark AGujas, BojanSobeh, MansourGulati, VandanaSomssich, MarcGuy, Charles L.Song, GuoqingHackauf, BerndSorieul, MathiasHammerschmidt, RaySpiegler, VerenaHammes, UlrichSpringer, PatriciaHano, ChristopheStadler, MarcHarris, Philip J.Strati, Irini F.Hashiguchi, AkikoSuleria, HafizHatano, TsutomuSweeney, JonHay, AngelaSymkal, PetrHeaney, LiamSyrpas, MichailHeilmann, MareikeTakeshi, MikiHeiskanen, JuhaTaranto, FrancescaHelaly, SoleimanTattini, MassimilianoHerrero, BaudilioTedesco, IdoloHildreth, SherryTellez, Trinidad RuízHimeno, HyoutaTerzaghi, WilliamHiruma, KeiThammina, ChandraHo, HonhingThapa, SushilHoch, GünterThijs, SofieHolalu, SrinidhiThilmony, RogerHooykaas, Paul J.J.Thiruvengadam, MuthuHorgan, Finbarr G.Thomas, ElizabethHorim, LeeTomi, FélixHu, GuangganTorres-Martínez, LorenaHu, ZhenbinTorricelli, RenzoHuang, LeiTripodi, PasqualeHuggins, TrevisTsai, Mei-LinIschebeck, TillTsopmo, ApollinaireIslam, M. NurulTurgeon, RobertIwasaki, NaotoTytła, MalwinaJanga, MadhusudhanaUmehara, MikihisaJansen, MarcusUnderwood, WilliamJaradat, NidalUpadhyay, Rakesh K.Jarquin, DiegoUygun, SahraJarret, Robert L.Van Esse, WilmaJiang, Shu-YeVan Heusden, Adriaan WJohnson, KimVaughan, Martha MJones, HuwVaz, JosianaJordão, António ManuelVenäläinen, SallaJuliano, ClaudiaVenturi, FrancescaKaiser, EliasVerger, StéphaneKanaoka, MasahiroVicente, ClaudiaKatsuhara, MakiVolaire, FlorenceKeplinger, TobiasWalker, Berkley JamesKhallouki, FaridWang, KunKim, Ki HyunWardill, Hannah R.Kimbaris, Athanassios S.Wege, StefanieKirst, HenningWehling, PeterKnox, PaulWenkel, StephanKobayashi, Natsuko I.Wientjes, EmilieKolukisaoglu, ÜnerWiggins, GregKondo, YukiWilkinson, Mark D.Kopecký, DavidWu, KeqiangKosova, KlaraWu, QingyuKozaki, AkikoXiao, JunKozlov, MikhailXu, PengKumar, ManuYang, InhoKumpf, RobertYordanov, YordanKuo, Ping-ChungYoshioka, KeikoKurczynska, EwaZambrano-Wilson, Maria ClemenciaLachman, JaromirZamyatnin, Jr., Andrey A.Lahiri, SriyankaZarra, IgnacioLang, IngeborgZavafer, AlonsoLarena, InmaculadaZdunek, ArturLatosińska, JolantaZhou, BangjunLee, Sang-HanZuo, Ran

